# Modified Commando procedure using a double valve composite through an aorto-annulo-septotomy

**DOI:** 10.1093/icvts/ivad213

**Published:** 2024-01-05

**Authors:** Kanji Matsuzaki, Kisato Mitomi, Akito Imai, Masataka Sato, Yasunori Watanabe

**Affiliations:** Department of Cardiovascular Surgery, Hitachi General Hospital, Hitachi, Japan; Department of Cardiovascular Surgery, Hitachi General Hospital, Hitachi, Japan; Department of Cardiovascular Surgery, Hitachi General Hospital, Hitachi, Japan; Department of Cardiovascular Surgery, Hitachi General Hospital, Hitachi, Japan; Department of Cardiovascular Surgery, Hitachi General Hospital, Hitachi, Japan

**Keywords:** Commando procedure, Double valve replacement, Intervalvular fibrous body, Prosthetic valve endocarditis

## Abstract

Double valve replacement of aortic and mitral valves with intervalvular fibrous body reconstruction (Commando procedure) is a challenging operation. Particularly in redo surgery for prosthetic valve endocarditis, special techniques are needed for approaching and reconstructing the valve complex. We performed a modified Commando procedure using a double valve composite through an aorto-annulo-septotomy. This approach provided a good field of vision at the combined aortic and mitral annuli without incising the left atrial roof. The double valve composite with four-layer patch wings was useful for performing double valve replacement en bloc and aorto-annulo-septotomy closure serially. Using these techniques, we successfully performed the Commando procedure for complicated prosthetic valve endocarditis.

## INTRODUCTION

The Commando procedure is a challenging operation consisting of double valve replacement (DVR) of aortic and mitral valves with intervalvular fibrous body (IFB) reconstruction [1, 2]. Particularly in redo DVR for prosthetic valve endocarditis (PVE), special techniques may be needed in approaching both valves and reconstructing the IFB complex because of postoperative adhesion and infectious destruction [3, 4]. We report our modified Commando procedure using a double valve composite through an aorto-annulo-septotomy.

## CASE

A 52-year-old man (height 170 cm, weight 64 kg) had undergone DVR with mechanical prostheses [20-mm ATS AP360 (Medtronic Medical, Santa Rosa, CA, USA) and 25-mm SJM (St Jude Medical, St Paul, MN, USA)] for infectious endocarditis 4 years before. He was hospitalized with PVE that developed in those prostheses. Although he was treated effectively with antibiotics for 6 weeks, we performed redo DVR for deteriorating heart failure. Transoesophageal echocardiography revealed abnormal shunt flow appearing from the aortic annulus into the left atrial cavity (Video 1).

## SURGICAL TECHNIQUES

**Figure 1:**
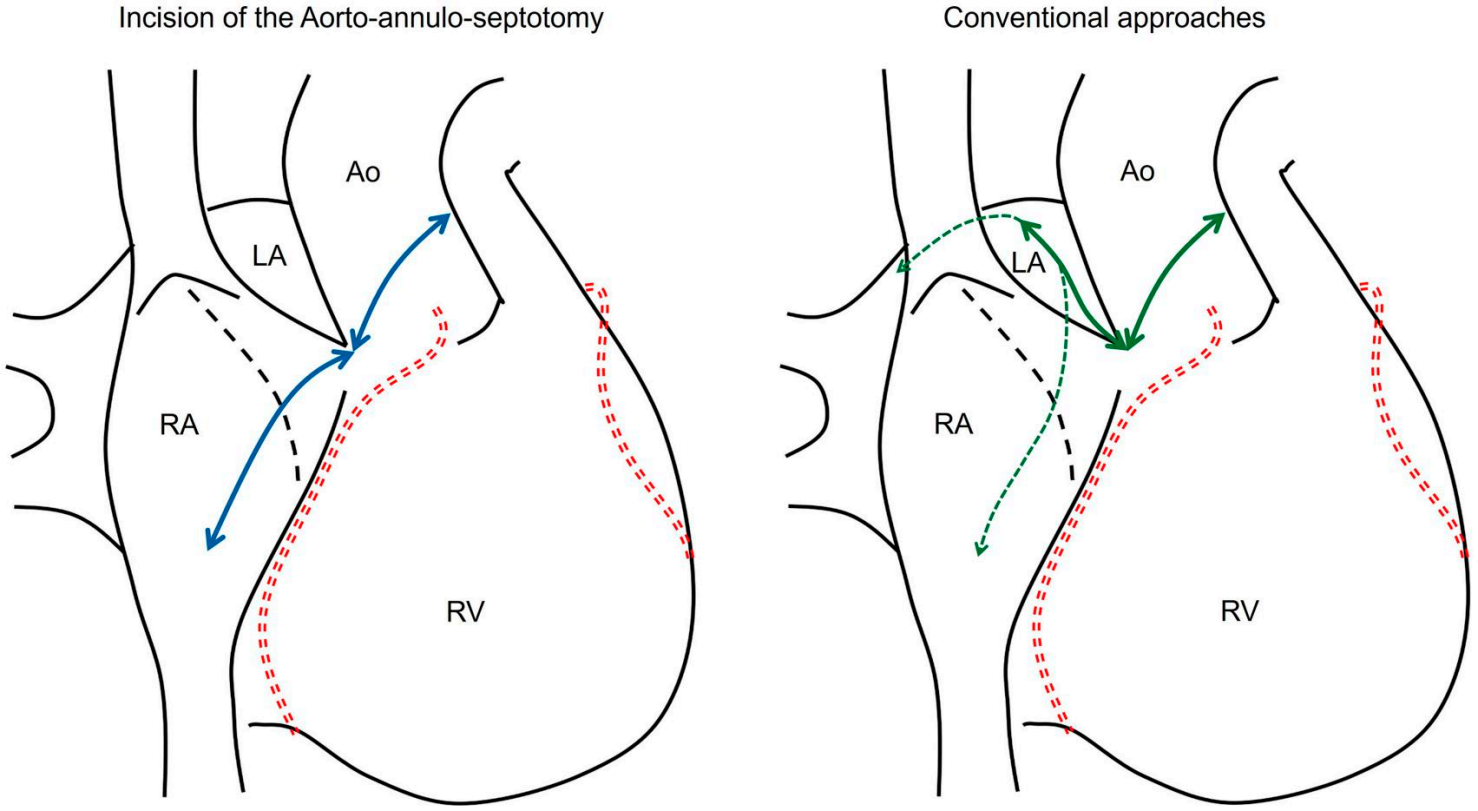
Schema of the aortic and right atrial incision of an aorto-annulo-septotomy compared with conventional approaches. Ao: aorta; LA: left atrium; RA: right atrium: RV: right ventricle.

**Figure 2:**
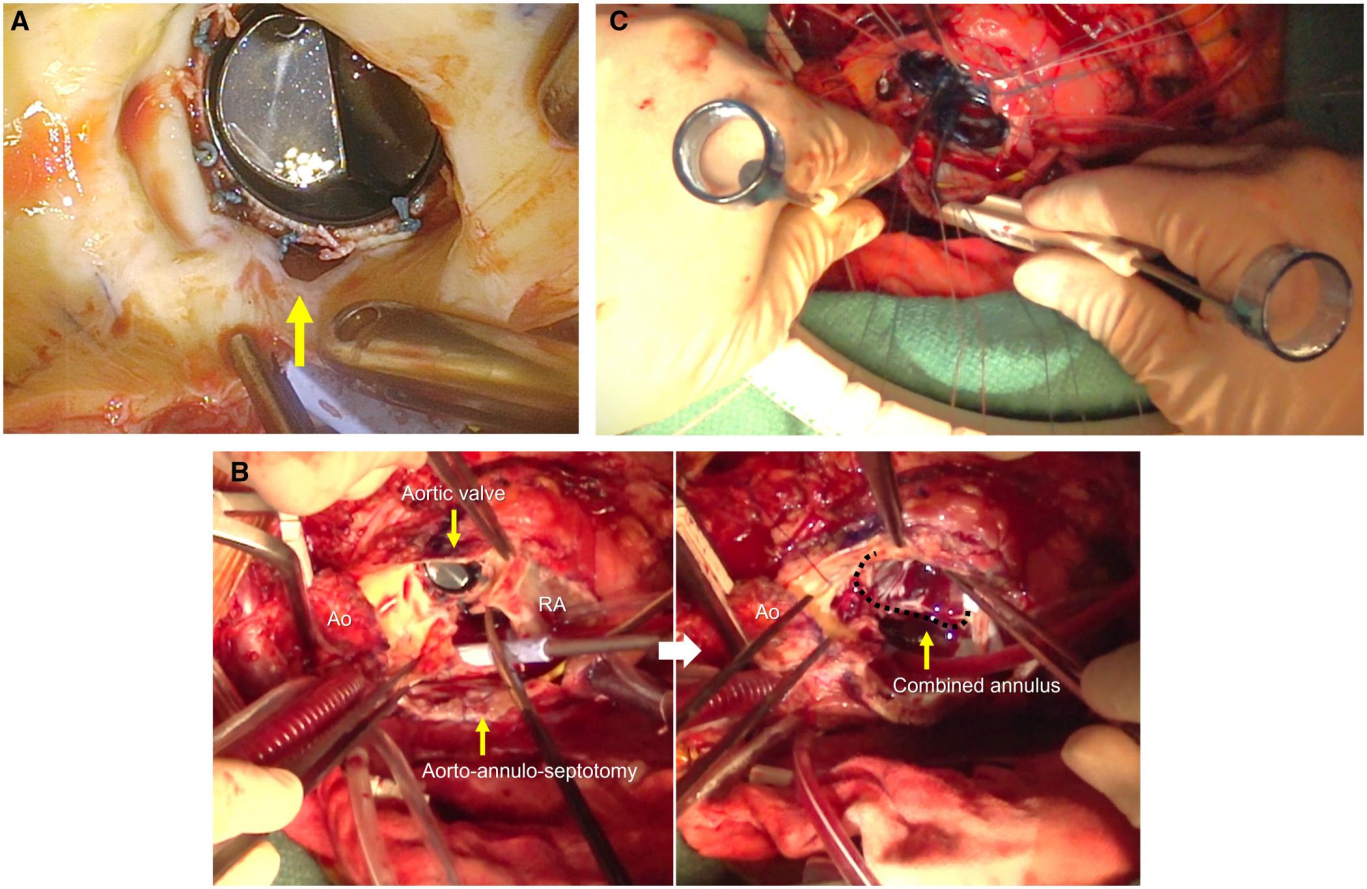
(**A**) Perforation of the intervalvular fibrous body at the noncoronary aortic annulus (yellow arrow). (**B**) The surgeon’s view of the aorto-annulo-septotomy and the combined annulus. (**C**) Sizing the combined annulus using 2 valve sizers. Ao: aorta; RA: right atrium.

Following a median resternotomy, cardiopulmonary bypass was performed with arterial perfusion via the femoral artery and bicaval drainage. After clamping the ascending aorta under ventricular fibrillation, we performed an oblique aortotomy down to the mid portion of the noncoronary sinus (Fig. 1; Video 2). Through the aortotomy, we found a 5-mm perforation of the IFB at the noncoronary aortic annulus (Fig. 2A). Myocardial protection was achieved by initial selective cardioplegia, and the right atrium was opened after snaring the vena cava. We maintained retrograde cardioplegia intermittently and added a vertical septotomy in the fossa ovalis. This interatrial septotomy was extended up towards the mid portion of the noncoronary sinus and connected to the oblique aortotomy (aorto-annulo-septotomy) (Fig. 2B). The damaged prostheses and IFB were removed by surgical scissors. As a result, the aorto-annulo-septotomy provided a good field of vision at the combined aortic and mitral annuli without incising the left atrial roof (Fig. 2B).

**Figure 3:**
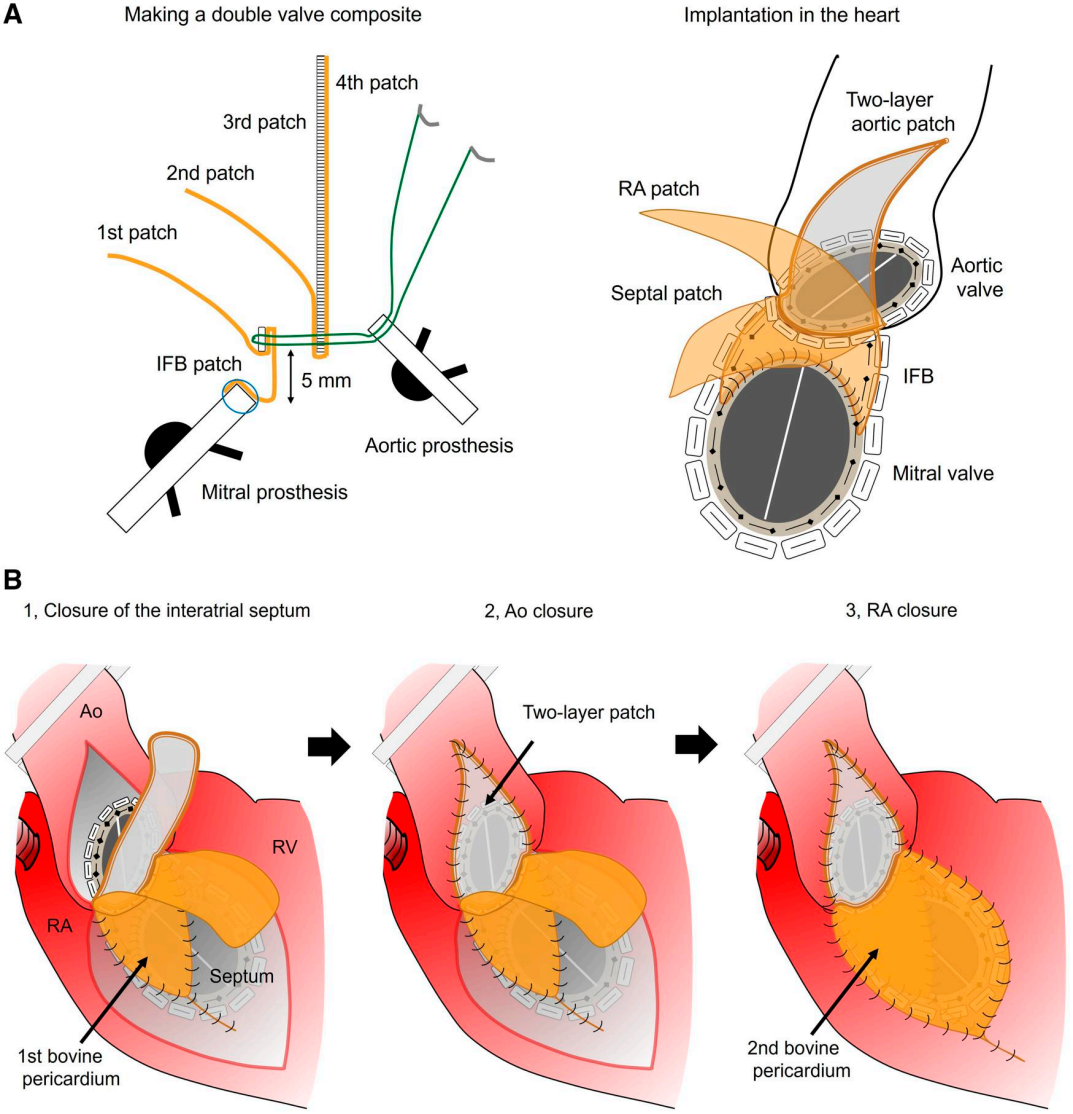
(**A**) Schema of making a double valve composite and implanting it in the heart. (**B**) Reconstruction of the interatrial septum, the right atrium and the aorta using the four-layer patch wings. Ao: aorta; IFB: intervalvular fibrous body; RA: right atrium: RV: right ventricle.

Following debridement and repair of the combined annulus, 22 everting mattress 2–0 polyester sutures were placed (Video 3). The hole size was measured precisely using 2 manufacturers’ valve sizers simultaneously (Fig. 2C). Based on these measurements, we determined the shape and size of a patch for the IFB. A double valve composite was made with 19-mm Regent and 25-mm standard St Jude Medical mechanical prostheses and patches (Fig. 3A). In short, a sheet of bovine pericardium was tailored to a half circumference of the mitral prosthesis and sewn to its sewing ring using a running 4–0 polypropylene suture; a three-layer patch was made by sandwiching a Dacron patch between 2 sheets of bovine pericardium. The valved pericardium was folded along and moderately apart from the mitral prosthesis, and its ridge was sewn to the three-layer patch and onto about 1/3 of the sewing ring of the aortic prosthesis using 5 mattress 2–0 polyester sutures. That is, the distance of the reconstructing IFB between the 2 prostheses resulted in about 5 mm at the centre and 15 mm at both ends. Using the double valve composite with four-layer patch wings, we performed DVR en bloc and aorto-annulo-septotomy closure serially (Fig. 3B). The sewing rim was secured on the combined annulus using 22 mattress sutures placed first. From the mitral side, the first bovine pericardium of the four-layer patch wings was used to reconstruct the IFB and further to close the interatrial septum. The second patch of bovine pericardium was used to close the right atrial free wall. The two-layer composite patch, which consisted of the third patch of the Dacron sheet and the fourth patch of bovine pericardium, was used to close the aorta (Fig. 3A). We had no difficulty in achieving haemostasis, and cardiac function recovered well.

## POSTOPERATIVE COURSE

The patient received postoperative antibiotic treatment, and a permanent pacemaker was implanted due to an unstable conduction system. Transthoracic echocardiography revealed no abnormal function or paravalvular leakage of the double valve composite (Video 4). He was discharged 7 weeks postoperatively.

## DISCUSSION

The standard approach for the Commando procedure involves performing an oblique aortotomy, extending its proximal incision into the IFB and into the left atrial roof (Fig. 1) [3]. In complicated cases, some modified techniques are used to obtain good exposure of the IFB, such as transient division of the superior vena cava [1, 5] and an extended transseptal approach with incision of the left atrial roof [6, 7]. In the present case, we used an aorto-annulo-septotomy, which included an oblique aortotomy and an extended transseptal approach without incising the left atrial roof (Fig. 1). A similar technique was reported by Hassan *et al.* as a combined transaortic and transseptal atrial approach [8]. This approach provided a good field of vision at the combined aortic and mitral annuli from the front, even in redo surgery for PVE. We did not have to dissect the left atrial roof, which adhered strongly to the neighbouring tissues, thereby contributing to good haemostasis. On the other hand, an annulus incision close to the right fibrous trigone may have affected the conduction system [1]. To avoid interference with the major conduction pathway, we incised the aortic annulus moderately apart from the right fibrous trigone. Radical debridement of the damaged IFB would have led to conduction disorder.

In most Commando procedures reported in the literature, mitral valve replacement, IFB reconstruction and aortic valve replacement were performed separately [3]. In this study, we conducted those 3 steps simultaneously using a double valve composite. A similar technique was reported by Kitamura *et al.* as coupling valve grafting in 1983 [9]. They applied this technique primarily to aortic and mitral stenosis with small annuli. They also proposed the optimal size combinations of aortic and mitral prostheses for DVR [9]. One of those was 19 mm in the aortic position and 25 mm in the mitral position; these same sizes were used in the present case. A previous study reported that proper sizing and offsetting of the aortic and mitral prostheses are important in order to avoid obstructing the left ventricular outflow tract and compromising the valve-opening mechanism [8]. We measured both the aortic and mitral annuli using 2 valve sizers simultaneously to prevent prosthetic mismatch (Fig. 2C), and the double valve composite was made with an optimal pair of prostheses. Although valvular prostheses have developed remarkably over the past 40 years, their optimal size combinations may not have changed because prostheses for both positions have evolved in the same manner. To prevent patient–prosthesis mismatch, small prostheses with increased geometric orifice area are available today.

After the Commando procedure for PVE, paravalvular leakage is a major concern at follow-up [10]. Our procedure may be helpful in preventing patch dehiscence of the reconstructed IFB because the double valve composite has been sewn before it is implanted. Moreover, the equally balanced mattress sutures securing the composite on the combined annulus may reduce the risk of paravalvular leakage by attenuating annulus distortion. We also used the four-layer patch wings of the composite to serially close the aorto-annulo-septotomy. When closing the interatrial septum and the right atrial free wall, bovine pericardia were easily tailored and sewn to each defect. When closing the aortic root, a two-layer combination of bovine pericardium and Dacron patch contributed to good haemostasis. The composite patch was also sufficiently strong to repair the aortic root. Although there is as yet a lack of convincing evidence, we expect that its durability may be superior to that of bovine pericardium alone.

We successfully performed our modified Commando procedure for PVE. An aorto-annulo-septotomy provided a good field of vision at the combined aortic and mitral annuli without incising the left atrial roof. The double valve composite with four-layer patch wings was useful for performing DVR en bloc and aorto-annulo-septotomy closure serially. Hassan *et al.* performed the former technique but not the latter, and Kitamura *et al.* performed the latter but not the former [8, 9]. This is the first report of the integrated performance of these two techniques. However, this procedure cannot be applied to patients in whom the infection and abscess have spread into the right and left coronary sinuses. This procedure is also still complex with a long cross-clamp time. Because the present case was our first experience, we did not think about making a double valve composite before aortic cross-clamping. It might have been possible to estimate the size of the new prostheses from the old ones and from preoperative images. We plan to consider that strategy next time.

## Data Availability

All relevant data are within the manuscript.
